# Supplementary material for: A Statistical Model for In Vivo Neuronal Dynamics
Source: PLoS One. 2015 Nov 16;10(11):e0142435. doi: 10.1371/journal.pone.0142435 (PMC4646699; doi:10.1371/journal.pone.0142435)
Supplement: S1 Text — Information on Discrete Fourier Transforms, circulant matrices, the circulant approximation, sampling from GPs using Fast Fourier Transforms, details of the optimization scheme, gradients and hessians used for the optimization. (PDF) [file pone.0142435.s001.pdf]

Supplementary Text

A statistical model for in vivo neuronal dynamics

Simone Carlo Surace[1,2,*], Jean-Pascal Pfister[2]
1 Department of Physiology, University of Bern, Bern, Switzerland
2 Institute of Neuroinformatics, University of Zurich and ETH Zurich, Zurich, Switzerland
*** E-mail: surace@pyl.unibe.ch**

Discrete Fourier Transform

In the following and in the main text, we denote discrete Fourier transforms of vectors of length n by a hat. The Fourier transformed vector is again of length n and can be formally expressed as

$$\hat{v} = \mathbb{F}v, \quad (\mathbb{F}_n)_{ij} = e^{\frac{2\pi I(i-1)(j-1)}{n}}, \quad i,j = 1,...,n, \tag{S1}$$

where I denotes the imaginary unit. In practice, discrete Fourier transforms are not actually computed by matrix multiplication, but by means of a Fast Fourier Transform (FFT) algorithm.

Circulant matrices

In order to reduce the computational complexity of the likelihood estimation, we approximate the auto-covariance matrix K (which is a Toeplitz matrix K) with a circulant matrix C. By definition a circulant matrix can be expressed as

$$C_{ij} = c_{(i-j \bmod n)+1} \tag{S2}$$

we write $C = C_n(c)$. All circulant matrices of dimension n can be diagonalized by the unitary discrete Fourier transform matrix $\mathbb{U} = \frac{1}{\sqrt{n}}\mathbb{F}_n$:

$$C_n(c) = \mathbb{U}_n^\dagger \mathrm{diag}(\mathbb{F}_n c)\mathbb{U}_n = \mathbb{U}_n^\dagger \mathrm{diag}(\hat{c})\mathbb{U}_n, \tag{S3}$$

where $\dagger$ is the conjugate transpose. This implies that $\hat{c}$ is the vector of eigenvalues of C, and it is a vector with real entries. This makes calculation of inverse and determinant of C extremely cheap, as is multiplication of C^{-1} by a vector $x \in \mathbb{R}^n$, which simplifies to

$$C_n^{-1}(c)x = \frac{1}{n}\mathbb{F}_n^\dagger \left(\frac{\hat{x}}{\hat{c}}\right) \tag{S4}$$

where the vector in brackets is the component-wise quotient of the vectors $\mathbb{U}_n x$ and $\mathbb{F}_n c$.

Circulant approximation

The task is now to find a circulant matrix which is as close as possible to the covariance matrix K. This can be formalized as the following minimization problem:

$$C = \underset{D \text{ circulant}}{\mathrm{argmin}}\ D_{\mathrm{KL}}\left(\mathcal{N}(m,K)||\mathcal{N}(m,D)\right), \quad \forall m \tag{S5}$$

where $\mathcal{N}$ denotes a multivariate Gaussian with specified mean vector and covariance matrix. This problem has the unique solution

$$c_i = \frac{1}{n}\left\{(n-i+1)k_i + (i-1)k_{n-i+2}\right\}, \quad 1 \le i \le n, \quad k_{n+1} \equiv 0 \tag{S6}$$

Proof: The Kullback-Leibler divergence between two Gaussians is given by

$$D_{\mathrm{KL}}\left(\mathcal{N}(m,K)||\mathcal{N}(m,C)\right) = \frac{1}{2}\left[\mathrm{tr}(C^{-1}K) - \log\det(C^{-1}K)\right] - \frac{n}{2} \tag{S7}$$

We have

$$C^{-1} = \mathbb{U}_n^\dagger \mathrm{diag}\left(\frac{1}{\hat{c}}\right)\mathbb{U}_n, \qquad \det C^{-1} = \prod_{i=1}^{n}\frac{1}{\hat{c}_i} \tag{S8}$$

and hence

$$D_{\mathrm{KL}}\left(\mathcal{N}(m,K)||\mathcal{N}(m,C)\right) = \frac{1}{2}\sum_{i=1}^{n}\left[\frac{(\mathbb{U}_n K \mathbb{U}_n^\dagger)_{ii}}{\hat{c}_i} + \log\hat{c}_i\right] + \mathrm{const.} \tag{S9}$$

where the constant does not depend on c. We obtain the derivative

$$\frac{\partial}{\partial c_i} D_{\mathrm{KL}}\left(\mathcal{N}(m,K)||\mathcal{N}(m,C)\right) = \frac{1}{2\hat{c}_i}\left[1 - \frac{(\mathbb{U}_n K \mathbb{U}_n^\dagger)_{ii}}{\hat{c}_i}\right] = 0 \tag{S10}$$

and therefore, at the stationary point we have

$$\begin{aligned}
\hat{c}_i &= (\mathbb{U}_n K \mathbb{U}_n^\dagger)_{ii} \\
c_i &= \frac{1}{n^2}\sum_{j,l,m=1}^{n} (\mathbb{F}_n^\dagger)_{ij}(\mathbb{F}_n)_{jl} K_{lm} (\mathbb{F}_n^\dagger)_{mj} \\
&= \frac{1}{n^2}\sum_{j,l,m=1}^{n} K_{lm}\exp\left[\frac{2\pi I}{n}\left(-(i-1)(j-1) + (j-1)(l-1) - (m-1)(j-1)\right)\right] \\
&= \frac{1}{n^2}\sum_{j,l,m=1}^{n} k_{|l-m|+1}\exp\left[\frac{2\pi I}{n}(j-1)(l-m+1-i)\right]
\end{aligned} \tag{S11}$$

The sum of roots of unity over j only gives a non-zero value if the integer $q = l - m + 1 - i$ is a multiple of n. Since q has a maximum of $q = n-1$ when $l = n, m = i = 1$ and a minimum of $q = 2 - 2n$ when $l = 1, m = i = n$, only $q = 0$ and $q = -n$ are eligible. Hence,

$$\begin{aligned}
c_i &= \frac{1}{n^2}\sum_{l,m=1}^{n} n k_{|l-m|+1}\left(\delta_{0,l-m+1-i} + \delta_{-n,l-m+1-i}\right) \\
&= \frac{1}{n}\sum_{r=-n+1}^{n-1} (n-|r|) k_{|r|+1}\left(\delta_{i-1,r} + \delta_{i-1,n+r}\right) \\
&= \frac{1}{n}\left\{(n-i+1)k_i + (i-1)k_{n-i+2}\right\}, \quad k_{n+1} \equiv 0
\end{aligned} \tag{S12}$$

The second equality is obtained by reparametrizing $r = l - m$. □

Sampling using FFTs

In order generate a sample u of length n from a multivariate Gaussian with mean vector m and circulant covariance matrix $C = C_n(c)$, one generates a zero-mean white noise vector x with unit variance and then uses FFTs to compute u, i.e.

$$u = m + \frac{1}{n}\mathbb{F}_n^\dagger\left[(\hat{c})^{1/2}\,\hat{x}\right] = m + \frac{1}{n}C_n\left(\mathbb{F}_n^\dagger\,(\hat{c})^{1/2}\right)x \tag{S13}$$

as the following calculation shows, the covariance comes out correctly

$$\begin{aligned}\left\langle (u-m)(u-m)^T \right\rangle &= \frac{1}{n^2} C_n \left(\mathbb{F}_n^\dagger \left(\hat{c}\right)^{1/2} \right) \left\langle x x^T \right\rangle C_n \left(\mathbb{F}_n^\dagger \left(\hat{c}\right)^{1/2} \right)^T \\ &= \frac{1}{n} C_n \left(\mathbb{F}_n^\dagger \hat{c} \right) = C_n(c) = C\end{aligned} \tag{S14}$$

Optimization method

The optimization scheme used is a quasi-Newton method, where the vector of parameters Θ is updated according to

$$\Theta^{(k+1)} = \Theta^{(k)} - B^{(k)} \nabla f \left(\Theta^{(k)} \right) \tag{S15}$$

where f is the function to be minimized (e.g. $-\log p(u_{\text{som}}, s)$), ∇f is the gradient, and the matrix B is chosen to be

$$B^{(k)} = \begin{cases} H_f^{-1}\left(\Theta^{(k)}\right), & \text{if } H_f\left(\Theta^{(k)}\right) \text{ positive definite} \\ \gamma^{(k)} G^{-1}, & \text{else} \end{cases} \tag{S16}$$

Where H_f is the Hessian of f, $\gamma^{(k)}$ denotes a learning rate, and G is a metric tensor on the parameter space which is used to rescale the parameters to lie in similar ranges. The learning rate $\gamma^{(k)} < 0$ is increased when the previous step was successful (typically, by 10 percent), and reduced when the optimizer either runs into boundaries of the admissible parameter region or increases the value of the function (we used a reduction by a factor of 2).

Below, we derive the formulae for the gradient and Hessian required for the optimization. The derivations hold for the case where the GP covariance function k is parametrized arbitrarily by θ_i and the spike-shape kernel and adaptation kernel are given by linear combinations of basis functions $\alpha^{(k)}$, $k = 1, ..., n_a$ and $\eta^{(k)}$, $k = 1, ..., n_w$ respectively.

Gradient

The likelihood function has the form

$$\log p(u_{\text{som}}, s) = \sum_{i=1}^{n} \left[-\frac{1}{2} \log(2\pi \hat{c}_i) - \frac{1}{2n} \frac{|\hat{u}_i|^2}{\hat{c}_i} + s_i \log q_i + (1 - s_i) \log\left[1 - q_i\right] \right], \tag{S17}$$

where

$$q_i = \Delta t e^{\beta u_i + A_i + \log r_0} \tag{S18}$$

is the probability of a spike in bin i and depends on all parameters except the ones that parametrize the covariance function k. The membrane potential is given implicitly by

$$u = u_{\text{som}} - u_r - \alpha * s. \tag{S19}$$

It is worthwhile to write the derivatives of $\log p$ in the following form:

$$d \log p(u_{\text{som}}, s) = \sum_{i=1}^{n} \left[-\frac{1}{2} \left(\frac{1}{\hat{c}_i} - \frac{1}{n} \frac{|\hat{u}_i|^2}{\hat{c}_i^2} \right) d\hat{c}_i - \frac{1}{n \hat{c}_i} \Re\left\{ \hat{u}_i^* d\hat{u}_i \right\} + \frac{s_i - q_i}{q_i(1 - q_i)} dq_i \right], \tag{S20}$$

Now, let us evaluate all the terms one by one. The Fourier transform of the circulant covariance c only depends on the GP kernel parameters θ, i.e.

$$d\hat{c}_i = \frac{\partial \hat{c}_i}{\partial \theta_k} d\theta_k = \sum_{k=1}^{n_k} \left(\widehat{\frac{\partial c}{\partial \theta_k}} \right)_i d\theta_k. \tag{S21}$$

Let us turn to the q terms next. Their differential is

$$dq_i = \frac{\partial q_i}{\partial u_i} du_i + \frac{\partial q_i}{\partial A_i} dA_i + \frac{\partial q_i}{\partial r_0} dr_0 + \frac{\partial q_i}{\partial \beta} d\beta = q_i(\beta du_i + dA_i + d\log r_0 + u_i d\beta), \tag{S22}$$

where by (S19) and using the fact that α is a linear combination of basis kernels $\sum_{k=1}^{n_\alpha} a_k \alpha^{(k)}$

$$du_i = -du_r - \sum_{k=1}^{n_\alpha} S_i^{(k)} da_k, \quad S_i^{(k)} = (\alpha^{(k)} * s)_i. \tag{S23}$$

Moreover, A_i is also a linear combination, so

$$dA_i = \sum_{k=1}^{n_\eta} A_i^{(k)} dw_k, \quad A_i^{(k)} = (\eta^{(k)} * s)_i. \tag{S24}$$

Therefore (S22) can be written as

$$dq_i = q_i \left(-\beta du_r - \beta \sum_{k=1}^{n_\alpha} S_i^{(k)} da_k + \sum_{k=1}^{n_\eta} A_i^{(k)} dw_k + d\log r_0 + u_i d\beta \right), \tag{S25}$$

Lastly, by (S23) we also have

$$d\hat{u}_i = -n\delta_{1i} du_r - \sum_{k=1}^{n_\alpha} \hat{S}_i^{(k)} da_k. \tag{S26}$$

Using

$$v_i = \frac{s_i - q_i}{1 - q_i}, \tag{S27}$$

all the previous results can be regrouped to yield

$$\begin{aligned} d\log p(u_{\text{som}}, s) = \sum_{i=1}^{n} \Bigg[-\frac{1}{2} \left(\frac{1}{\hat{c}_i} - \frac{1}{n} \frac{|\hat{u}_i|^2}{\hat{c}_i^2} \right) \sum_{k=1}^{n_k} \left(\widehat{\frac{\partial \hat{c}_i}{\partial \theta_k}} \right)_i d\theta_k \\ + \sum_{k=1}^{n_\alpha} \left(\frac{1}{n\hat{c}_i} \Re \left\{ \hat{u}_i^* \hat{S}_i^{(k)} \right\} - \beta S_i^{(k)} v_i \right) da_k \\ + \sum_{k=1}^{n_\eta} A_i^{(k)} v_i dw_k \\ + v_i d\log r_0 \\ + u_i v_i d\beta \\ + \left(\frac{\delta_{1i}}{\hat{c}_i} \Re \left\{ \hat{u}_i \right\} - \beta v_i \right) du_r \Bigg], \end{aligned} \tag{S28}$$

Hessian

For the Hessian, we mainly need the following

$$-\frac{1}{2} d \left(\frac{1}{\hat{c}_i} - \frac{1}{n} \frac{|\hat{u}_i|^2}{\hat{c}_i^2} \right) = -\frac{1}{2} \left(\frac{1}{\hat{c}_i^2} - \frac{2}{n} \frac{|\hat{u}_i|^2}{\hat{c}_i^3} \right) d\hat{c}_i + \frac{1}{n\hat{c}_i^2} \Re \left\{ \hat{u}_i^* d\hat{u}_i \right\}, \tag{S29}$$

$$d\left(\frac{1}{n\hat{c}_i}\Re\left\{\hat{u}_i^*\hat{S}_i^{(k)}\right\}\right) = -\frac{1}{n\hat{c}_i^2}\Re\left\{\hat{u}_i^*\hat{S}_i^{(k)}\right\}d\hat{c}_i + \frac{1}{n\hat{c}_i}\Re\left\{\hat{S}_i^{*(k)}d\hat{u}_i\right\}, \tag{S30}$$

$$dv_i = d\left(\frac{s_i - q_i}{1 - q_i}\right) = \frac{s_i - 1}{(1-q_i)^2}dq_i = \frac{\xi_i}{q_i}dq_i, \tag{S31}$$

where

$$\frac{\xi_i}{q_i} = \frac{s_i - 1}{(1-q_i)^2}. \tag{S32}$$

The components of the Hessian matrix are computed as follows

$$\frac{\partial^2 \log p(u_{\text{som}}, s)}{\partial\theta_k\partial\theta_l} = -\frac{1}{2}\sum_{i=1}^{n}\left\{\frac{\partial^2\hat{c}_i}{\partial\theta_k\partial\theta_l}\left[\frac{1}{\hat{c}_i} - \frac{1}{n}\left|\frac{\hat{u}_i}{\hat{c}_i}\right|^2\right] - \frac{\partial\hat{c}_i}{\partial\theta_k}\frac{\partial\hat{c}_i}{\partial\theta_l}\left[\frac{1}{\hat{c}_i^2} - \frac{2}{n}\frac{|\hat{u}_i|^2}{\hat{c}_i^3}\right]\right\}, \tag{S33}$$

$$\frac{\partial^2 \log p(u_{\text{som}}, s)}{\partial\theta_k\partial a_l} = -\frac{1}{n}\sum_{i=1}^{n}\frac{\partial\hat{c}_i}{\partial\theta_k}\Re\left\{\frac{\hat{u}_i^*\hat{S}_i^{(l)}}{\hat{c}_i^2}\right\}, \tag{S34}$$

$$\frac{\partial^2 \log p(u_{\text{som}}, s)}{\partial\theta_k\partial u_r} = -\frac{\partial\hat{c}_1}{\partial\theta_k}\Re\left\{\frac{\hat{u}_1}{\hat{c}_1^2}\right\}, \tag{S35}$$

$$\frac{\partial^2 \log p(u_{\text{som}}, s)}{\partial\theta_k\partial w_k} = \frac{\partial^2 \log p(u_{\text{som}}, s)}{\partial\theta_k\partial r_0} = \frac{\partial^2 \log p(u_{\text{som}}, s)}{\partial\theta_k\partial\beta} = 0, \tag{S36}$$

$$\frac{\partial^2 \log p(u_{\text{som}}, s)}{\partial a_k\partial a_l} = \sum_{i=1}^{n}\left[-\frac{1}{n}\Re\left\{\frac{\hat{S}_i^{*(k)}\hat{S}_i^{(l)}}{\hat{c}_i}\right\} + \beta^2 S_i^{(k)}S_i^{(l)}\xi_i\right], \tag{S37}$$

$$\frac{\partial^2 \log p(u_{\text{som}}, s)}{\partial a_k\partial w_l} = -\beta\sum_{i=1}^{n}S_i^{(k)}A_i^{(l)}\xi_i, \tag{S38}$$

$$\frac{\partial^2 \log p(u_{\text{som}}, s)}{\partial a_k\partial u_r} = -\Re\left\{\frac{\hat{S}_1^{(k)}}{\hat{c}_1}\right\} + \beta^2\sum_{i=1}^{n}S_i^{(k)}\xi_i, \tag{S39}$$

$$\frac{\partial^2 \log p(u_{\text{som}}, s)}{\partial a_k\partial\log r_0} = -\beta\sum_{i=1}^{n}S_i^{(k)}\xi_i, \tag{S40}$$

$$\frac{\partial^2 \log p(u_{\text{som}}, s)}{\partial a_k\partial\beta} = -\beta\sum_{i=1}^{n}S_i^{(k)}\xi_i u_i, \tag{S41}$$

$$\frac{\partial^2 \log p(u_{\text{som}}, s)}{\partial w_k\partial w_l} = \sum_{i=1}^{n}A_i^{(k)}A_i^{(l)}\xi_i, \tag{S42}$$

$$\frac{\partial^2 \log p(u_{\text{som}}, s)}{\partial w_k\partial u_r} = -\beta\sum_{i=1}^{n}A_i^{(k)}\xi_i, \tag{S43}$$

$$\frac{\partial^2 \log p(u_{\text{som}}, s)}{\partial w_k\partial\log r_0} = \sum_{i=1}^{n}A_i^{(k)}\xi_i, \tag{S44}$$

$$\frac{\partial^2 \log p(u_{\text{som}}, s)}{\partial w_k\partial\beta} = \sum_{i=1}^{n}A_i^{(k)}\xi_i u_i, \tag{S45}$$

$$\frac{\partial^2 \log p(u_{\mathrm{som}}, s)}{\partial u_r^2} = -\frac{n}{\hat{c}_1} + \beta^2 \sum_{i=1}^{n} \xi_i, \tag{S46}$$

$$\frac{\partial^2 \log p(u_{\mathrm{som}}, s)}{\partial u_r \partial \log r_0} = -\beta \sum_{i=1}^{n} \xi_i, \tag{S47}$$

$$\frac{\partial^2 \log p(u_{\mathrm{som}}, s)}{\partial u_r \partial \beta} = -\sum_{i=1}^{n} \left[v_i + \beta u_i \xi_i \right], \tag{S48}$$

$$\frac{\partial^2 \log p(u_{\mathrm{som}}, s)}{\partial (\log r_0)^2} = \sum_{i=1}^{n} \xi_i, \tag{S49}$$

$$\frac{\partial^2 \log p(u_{\mathrm{som}}, s)}{\partial \log r_0 \partial \beta} = \sum_{i=1}^{n} u_i \xi_i, \tag{S50}$$

$$\frac{\partial^2 \log p(u_{\mathrm{som}}, s)}{\partial \beta^2} = \sum_{i=1}^{n} u_i^2 \xi_i. \tag{S51}$$

The components as given by equations (33-51) are then combined to form the Hessian matrix H_f, which is used in Eq. (S16).
