# Supplementary figures and images for: A Statistical Model for In Vivo Neuronal Dynamics
Source: PLoS One. 2015 Nov 16;10(11):e0142435. doi: 10.1371/journal.pone.0142435 (PMC4646699; doi:10.1371/journal.pone.0142435)

A

 $\mathcal{D}_3$  data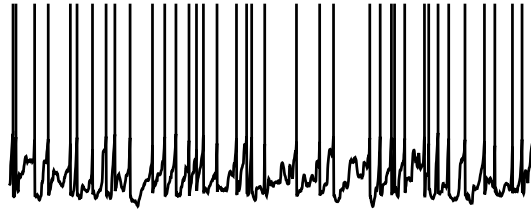

B

 $\mathcal{D}_3$  simulation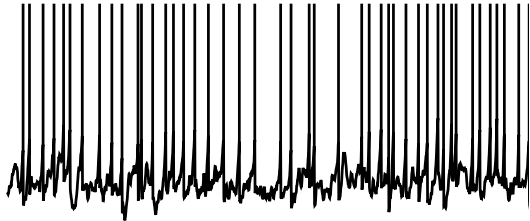

C

 $\mathcal{D}_4$  data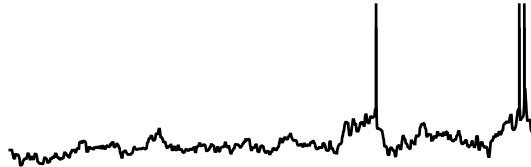

D

 $\mathcal{D}_4$  simulation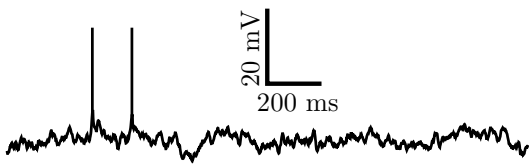

20 mV  
200 ms

Supplement: S1 Fig — Comparison of in vivo and artificial data snippets for datasets D3 and D4, analogous to Fig 3G and 3H. The scale (shown on panel D) is the same for all four panels. Vertical lines are drawn at the spiking times. (A) A 2-second sample of in vivo activity from dataset D3 (Zebra Finch HVC). (B) Artificial data sampled from AGAPE with parameters learned from dataset D3. (C) A 2-second sample of in vivo activity from dataset D4 (mouse visual cortex). (D) Artificial data sampled from AGAPE with parameters learned from dataset D4. (PDF) [file pone.0142435.s002.pdf]
